# Magnitude and associated factors of unintended pregnancy in Ethiopia: a multilevel analysis using 2016 EDHS data

**DOI:** 10.1186/s12884-020-03024-5

**Published:** 2020-05-29

**Authors:** Achamyeleh Birhanu Teshale, Getayeneh Antehunegn Tesema

**Affiliations:** Department of Epidemiology and Biostatistics, Institute of Public Health, College of Medicine and Health Sciences, University of Gondar, Gondar, Ethiopia

**Keywords:** Magnitude, Unintended pregnancy, Multilevel analysis, Ethiopia

## Abstract

**Background:**

Unintended pregnancy has become a significant public health and reproductive health problem that has had a substantial and appreciable adverse impact on mother, child, and the general public. Despite the paramount negative effects of unintended pregnancy, many pregnancies are unintended in Ethiopia. Therefore, this study aimed to determine the prevalence and associated factors of unintended pregnancy in Ethiopia.

**Methods:**

This study was based on the nationally representative 2016 Ethiopian Demographic and Health Survey data. We used a total weighted sample of 7590 reproductive-aged women who gave birth in the 5 years preceding the survey. A multi-level logistic regression analysis was used to account for the hierarchal nature of the DHS data. In the multivariable multilevel analysis, those variables with *p*-value < 0.05 were considered to be significantly associated with unintended pregnancy.

**Result:**

The prevalence of unintended pregnancy was 26.6% [95%CI: 25.6, 27.6]. In the multivariable multilevel logistic regression analysis; individual level variables such as being in the age group 20 to 34 [adjusted odds ratio (AOR) = 0.57; 95%CI: 0.41, 0.79] and 35 to 49 [AOR = 0.68; 95%CI: 0.47, 0.97], being follower of Muslim religion [AOR = 0.73; 95%CI: 0.60, 0.88], being married [AOR = 0.46; 95%CI: 0.37–0.58], household size of four to six [AOR = 1.38; 95%CI: 1.10, 1.69] and seven and above [AOR = 1.54; 95%CI: 1.20, 1.99], and being multiparous [AOR = 1.36; 95%CI: 1.10, 1.69] and grand multiparous [AOR = 1.92; 95%CI: 1.47, 2.52] were significantly associated with unintended pregnancy. Among community level variables; being living in large central [AOR = 2.56; 95%CI: 2.06, 3.17] and metropolitan regions [AOR = 1.91; 95%CI: 1.44, 2.53] were significantly associated with unintended pregnancy.

**Conclusion:**

In this study the prevalence of unintended pregnancy was high. Maternal age, religion, marital status, household size, parity, and region were the most important factors associated with unintended pregnancy. Special attention should, therefore, be given to younger, single, multiparous and grand multiparous women, and not follower of Muslim religion as well as mothers from large central and metropolitan regions in terms of increasing accessibility and affordability of maternal health services, which could minimize unintended pregnancy.

## Background

Unintended pregnancy is a pregnancy that is either unwanted (occurred when no children or no more children were desired) or mistimed (occurred earlier than desired) for at least one of the couples [1, 2]. Unintended pregnancy has been a major or troubling public health and reproductive health issue imposing a great and appreciable adverse consequence to the mother, child, and the public in general [3]. Mothers with unintended pregnancy are at risk of many devastating complications such as induced abortion to the extent of causing maternal death, higher crime rates, maternal depression, and parenting as well as family stress, reduced workforce efficiency, and reduced academic achievement [3–7]. In addition, mothers with unintended pregnancies are more likely to be careless on this pregnancy and might have late ante-natal care initiation and decreased delivery service utilization [8].

Abortion is a serious and devastating complication of unintended pregnancy in which one third (30.4%) of unintended pregnancies end up with abortion [6]. Another study showed that the occurrence of abortion is the primary consequence of unintended pregnancy in which about half of all unintended pregnancies end with abortion and this abortion comes with many complications directly related to the procedure such as hemorrhage, uterine perforation, cervical injury, and infection [8].

Overall, evidences revealed that unintended pregnancy is one of the most critical challenge facing the public health system and impose significant financial and social costs on society. In the world, between 2010 and 2014, an estimated 44% (62 unintended pregnancies per 1000 women) of pregnancies were unintended with a great discrepancy between developing (65 unintended pregnancies per 1000 women) and developed countries (45 per 1000 women) [9]. In sub-Saharan Africa, the overall prevalence of unintended pregnancy is 29% which ranges from 10.8% in Nigeria to 54.5% in Namibia [10]. Different studies conducted in Ethiopia also revealed that the prevalence of unintended pregnancy ranges from 13.7 to 41.5% [11–17].

Evidences have shown that socio-demographic factors such as; maternal age [15, 16, 18–22], level of education of the mother [19, 21–23], religion [17, 24, 25], marital status [12, 13, 16, 18, 26, 27], distance from the nearest health facility [13], parity [14, 16, 17, 21, 22], household size [12, 28, 29], income/wealth status [10, 19, 25], knowledge of ovulation cycle [14], knowledge of family planning [21], ever had of terminated pregnancy [15, 16, 20], residence [17, 21, 25] and region [22, 29] are the main factors associated with unintended pregnancy.

Tackling unintended pregnancy has a huge benefit in saving the costs of abortion and its complications, as well as reducing the morbidity and mortality of mothers and children [30]. Given the paramount negative consequences of unintended pregnancy for both the mother and the fetus, up to our knowledge, there was a limited study on unintended pregnancy that is based on nationally representative data in Ethiopia. Therefore, this study aimed to determine the prevalence and associated factors of unintended pregnancy. The finding from this study will give an insight for health care professionals and policymakers in understanding the burden of unintended pregnancy and its associated factors for setting possible interventions and ensure or deliver safe and reliable service to the reproductive age group women.

## Method

### Data source and population

This study was based on the nationally representative 2016 Ethiopian Demographic and Health Survey (EDHS) which was conducted from January 18, 2016, to June 27, 2016.

The survey used the Ethiopia Population and Housing Census (PHC) conducted in 2007 as a sampling frame. The frame is a complete list of 84,915 enumeration areas (EAs) created for the 2007 PHC. The sample used for the survey was stratified and selected using two stages. In the first stage, a total of 645 EAs (202 in urban areas and 443 in rural areas) were selected with probability proportional to EA size. In the second stage, 28 households per cluster were selected with an equal probability systematic selection. Detailed information was collected on issues related to the reproductive health (fertility and fertility preference, marriage, awareness and the use of family planning methods), adult and childhood morbidity and mortality as well as awareness and attitudes towards HIV/AIDS and other important public health issues from 16,650 households, 15,683 female respondents, and 12,688 male respondents. Further information regarding the sampling technique and questionnaire, in general about the survey, can be obtained from the EDHS 2016 report [31].

Our analysis was based on women’s data (IR data) set of EDHS 2016 which was accessed from the Measure demographic and health survey (DHS) program website (http://www.measuredhs.com). A total weighted sample of 7590 reproductive age women (15–49 years) who gave birth in the 5 years preceding the survey was used.

### Study variables

The dependent variable was unintended pregnancy which includes pregnancies that are wanted no more or wanted later. For this study, the independent variables were classified as individual level and community level variables. The individual-level variables were maternal age, maternal educational status, maternal occupation, marital status, religion, parity, household size, wealth status, media exposure, ever had of terminated pregnancy and knowledge of ovulation cycle. The community-level variables in this study were the perception of distance from the health facility, residence, and region. Some of the variables were re-coded to make suitable for the study such as wealth status, re-coded as poor = 0, middle = 1, rich = 2 whereby women in the poorest and poorer category were referred to as the “poor”; those who were above the poorer wealth category but not up to those in the high category were categorized as middle whilst those in the high wealth category (rich and richest) were recoded as rich. The other variable recoded was region in which; Afar, Somali, Benishangul, and Gambela as “small peripheral regions”, Tigray, Amhara, Oromia, and Sothern Nations Nationalities and Peoples Region (SNNPR) as “large central regions” and Harari, Dire Dawa, and Addis Ababa as “metropolitans”, based on their geopolitical features consistent with a previous study from Ethiopia [32]. Other important independent variables were also recoded or recategorized to make suitable for analysis.

### Data management and analysis

The extraction of data, coding, and analysis was done using Stata 14 software. The data were weighted with the available sample weight factor (v005) within the EDHS dataset to minimize the effect of sampling bias. A multi-level logistic regression analysis was used to account for the hierarchal nature of the DHS data and bivariable multilevel logistic regression analysis was performed to estimate the crude odds ratios at 95% confidence interval and those variables with *p*-value < 0.20 were considered for multivariable analysis. In the multivariable multilevel logistic analysis, those variables with *p*-value < 0.05 were considered to be significantly associated with unintended pregnancy.

After selecting variables for multivariable analysis, four models containing variables of interest were fitted. These models were model 1 without explanatory variables, model 2 which examined the effects of individual-level characteristics, model 3 examined the effect of community-level variables and model 4 which examined the effects of both individual and community level characteristics simultaneously. The random effect results were estimated using three different methods, the Intra-Class Correlation (ICC), median odds ratio (MOR), and proportional change in variance (PCV). Since these models were nested deviance was used to assess the model fitness.

## Results

### Background characteristics of respondents

Table 1 summarizes the background characteristics of the study participants. More than two-thirds of study participants were between 20 and 34 years of age with an overall mean age of 29.3(±6.8) years. The majority (63.1%) of respondents had no formal education and 43.5% of women had poor wealth status. Most, 93.7% of women were married while two-third of them had no media exposure. Regarding household size, half of the respondents were from a household size of four to six. The majority of the women were from rural areas and large peripheral regions.

**Table 1:** Background characteristics of respondents

Variables	Frequency	Percentage
Maternal age		
15–19	339	4.47
20–34	5292	69.72
35–49	1959	25.82
Maternal education		
No formal education	4791	63.12
Primary	2150	28.32
Secondary and above	649	8.55
Religion		
Orthodox	2882	37.97
Muslim	1652	21.76
Protestant	2824	37.21
Other	232	3.06
Marital status		
Married	7109	93.66
Not married	481	6.34
Occupation		
Working	4078	53.73
Not working	3512	46.27
Wealth index		
Poor	3306	43.55
Middle	1588	20.93
Rich	2696	35.52
Media exposure		
Yes	2621	34.53
No	4969	65.47
Household size		
1 to 3	1033	13.61
4 to 6	3889	51.23
7 and above	2668	35.26
Parity		
Primiparous	1434	18.90
Multiparous	3190	41.02
Grand multiparous	2966	39.08
Ever had of terminating a pregnancy		
Yes	6910	91.04
No	680	8.96
Distance from the health facility		
Big problem	4407	58.06
Not a big problem	3183	41.94
Residence		
Urban	969	12.77
Rural	6621	87.23
Region		
Large central	6900	90.90
Small peripheral	441	5.81
Metropolitan	249	3.28

### Prevalence of unintended pregnancy

**Fig. 1:**
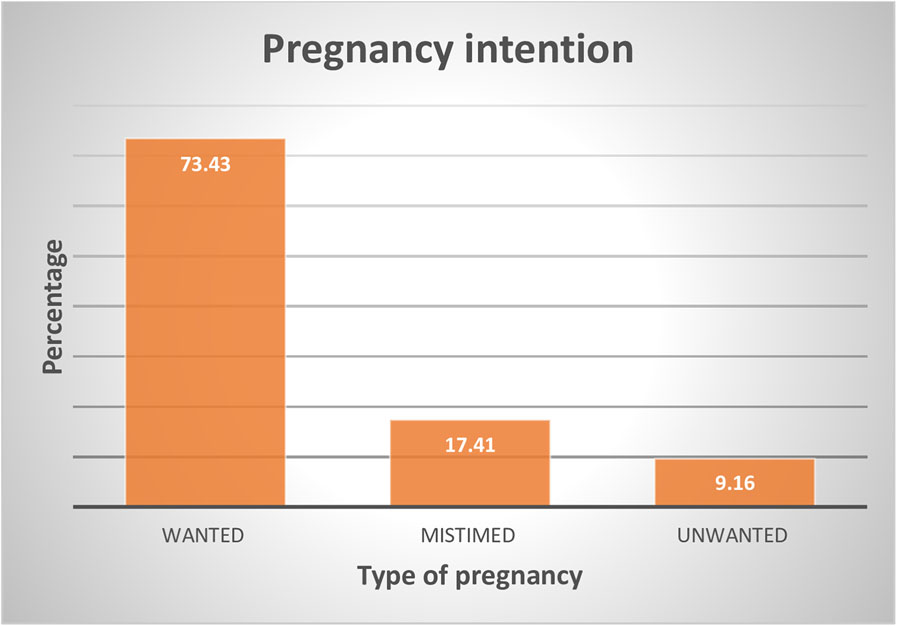
Prevalence of intended or wanted, unwanted and wanted but mistimed pregnancy in Ethiopia

In this study 73.4% [95%CI: 72.4, 74.4] of pregnancies were intended and 26.6% [95%CI: 25.6, 27.6] pregnancies were unintended (Fig. 1).

### Factors associated with unintended pregnancy

Table 2 showed the random effect model. In the null model, about 17% of the total variation on unintended pregnancy was occurred at the community level and is attributable to the community-level factors. The highest MOR value (2.17) in the null model revealed there was a variation of unintended pregnancy between clusters. Furthermore, the highest (36.4%) PCV in the final model (Model 4) indicates that 36.4% of the variation in unintended pregnancy across communities was explained by both individual and community level factors. The model fitness was checked using deviance and the model with the lowest deviance (Model 4) was the best-fitted model.

**Table 2:** Random effect (community-level clustering) in unintended pregnancy and model fitness

Parameter	Model 1	Model 2	Model 3	Model 4
MOR	2.17	2.00	1.87	1.85
PCV	Reff.	0.204	0.345	0.364
ICC	0.17	0.14	0.12	0.11
Deviance	7034.30	6815.46	6916.26	6740.00

Table 3 showed the fixed effects model. In the bivariable multilevel modeling, all of the independent (both individual level and community level variables), except knowledge of the ovulation cycle, had shown statistically significant association at a *p*-value of < 0.20. In the multivariable multilevel logistic regression analysis, the odds of unintended pregnancy was 43% [adjusted odds ratio (AOR) = 0.57; 95%CI: 0.41, 0.79] and 32% [AOR = 0.68; 95%CI: 0.47, 0.97] lower among mothers whose age was between 20 to 34 and 35 to 49 respectively as compared to those mothers whose age was 15 to 19 years. Mothers whose religion were Muslim had 27% [AOR = 0.73; 95%CI: 0.60, 0.88] lower odds of unintended pregnancy as compared to those mothers whose religion was Orthodox Christian. Regarding marital status, married mothers had 54% [AOR = 0.46; 95%CI: 0.37–0.58] lower odds of unintended pregnancy as compared to their counterparts. Respondents with a household size of four to six and seven and above had 1.38 [AOR = 1.38; 95%CI: 1.10, 1.69] and 1.54 [AOR = 1.54; 95%CI: 1.20, 1.99] times higher odds of unintended pregnancy respectively as compared with a household size of one to three. Multiparous and grand multiparous women had 1.36 [AOR = 1.36; 95%CI: 1.10, 1.69] and 1.92 [AOR = 1.92; 95%CI: 1.47, 2.52] times higher odds of unintended pregnancy respectively as compared to primiparous women. A woman who was living in large central regions and metropolitan regions had 2.56 [AOR = 2.56; 95%CI: 2.06, 3.17] and 1.91 [AOR = 1.91; 95%CI: 1.44, 2.53] times higher odds of unintended pregnancy respectively as compared to a woman from small peripheral regions.

**Table 3:** Multilevel logistic regression analysis of individual and community-level factors associated with unintended pregnancy in Ethiopia

Respondent characteristics	Model 1	Model 2 (AOR 95%CI)	Model 3 (AOR 95%CI)	Model 4 (AOR 95%CI)
*Individual-level and household level factors*				
Maternal age (years)				
15–19		1.00		1.00
20–34		0.59 [0.43–0.82]		0.57 [0.41–0.79]
35–49		0.71 [0.50–1.03]		0.68 [0.47–0.97]
Maternal education				
No formal education		0.88 [0.68–1.13]		0.84 [0.65–1.09]
Primary		1.15 [0.92–1.44]		1.19 [0.87–0.97]
Secondary and above		1.00		1.00
Religion				
Orthodox		1.00		1.00
Protestant		0.98 [0.79–1.12]		1.17 [0.95–1.45]
Muslim		0.54 [0.45–0.65]		0.73 [0.60–0.88]
Others		0.82 [0.51–1.32]		0.98 [0.61–1.57]
Marital status				
Not married		1.00		1.00
Married		0.46 [0.37–0.57]		0.46 [0.37–0.58]
Occupation				
Working		1.13 [0.98–1.29]		1.12 [0.98–1.28]
Not working		1.00		1.00
Household wealth				
Poor		1.00		1.00
Middle		1.15 [0.95–1.40]		1.01 [0.83–1.23]
Rich		1.21 [0.10–1.45]		1.03 [0.85–1.26]
Media exposure				
No		1.00		1.00
Yes		1.06 [0.90–1.24]		1.01 [0.86–1.19]
Household size				
1 to 3		1.00		1.00
4 to 6		1.41 [1.13–1.77]		1.38 [1.10–1.72]
7 and above		1.57 [1.22–2.02]		1.54 [1.20–1.99]
Parity				
Primiparous		1.00		1.00
Multiparous		1.33 [1.07–1.66]		1.36 [1.10–1.69]
Grand multiparous		1.87 [1.43–2.44]		1.92 [1.47–2.52]
Ever had of a terminated pregnancy				
Yes		1.22 [0.99–1.51]		1.19 [0.97–1.48]
No		1.00		1.00
*Community-level factors*				
Residence				
Urban			1.00	1.00
Rural			0.80 [0.64–1.01]	0.82 [0.62–1.07]
Region				
Metropolitan			1.81 [1.38–2.38]	2.56 [2.06–3.17]
Large central			2.92 [2.41–3.52]	1.91 [1.44–2.53]
Small peripheral			1.00	1.00
Distance from the health facility				
Big problem			1.00	1.00
Not a big problem			1.08 [0.93–1.24]	1.07 [0.93–1.24]

Note: *AOR* Adjusted Odds Ratio, *CI* Confidence Interval, *Other* Catholic, Traditional, Other

## Discussion

The study aimed to assess the prevalence and associated factors of unintended pregnancy in Ethiopia using EDHS 2016 data. According to the findings of this study, the estimated prevalence of unintended pregnancy in Ethiopia was 26.6% which is consistent with different studies done in Ethiopia [13, 17, 33]. This prevalence of unintended pregnancy in our study is less than studies done in Addis Ababa [27], Arsi Negele [16], Jimma [11], Botswana [34], and Ghana [22]. But our finding is higher than studies conducted in Tepi General Hospital Ethiopia [35], Belessa Woreda Ethiopia [15], a study based on 2011 EDHS data [14], Britain [23] and Cambodia [20]. This discrepancy might be due to the difference in the study population in which in the above studies (except studies in Ghana, 2011 EDHS data and Britain) pregnant women on antenatal care were their study subjects. Besides, most of the above studies were based on small sample size or they are mostly facility-based cross-sectional studies. The other possible explanation is due to the study period and different in socio-cultural characteristics as well as the difference in the availability and accessibility of maternal health services including family planning methods. Moreover, the discrepancy of our findings to that of a study in Britain might be due to Britain is one of a developed country in the world with accessible and organized maternal health services including family planning to prevent unintended pregnancy.

In multivariable multilevel logistic regression analysis maternal age, religion, marital status, household size, parity, and region were significantly associated with unintended pregnancy in Ethiopia. Consistent with different studies conducted in Pakistan [21], Nigeria [19], South Africa [36], Malawi [37], Kenya [18] and Cambodia [20], older mothers had lower odds of unintended pregnancy. This might be because of older women had higher knowledge on contraceptive methods to prevent unintended pregnancy and lower contraceptive failure rate [21, 38]. Additionally, this group of women might be more literate about the importance and accessibility of reproductive or maternal health services. Another plausible explanation is that older women are less likely to engage in risky sexual behaviors such as unprotected sexual intercourse and sex under the influence of drinking alcohol [39, 40].

In this study religion is another most important variable which significantly associated with unintended pregnancy. That is Muslim mothers were less likely to report having an unintended pregnancy as compared to orthodox Christian mothers. This finding is similar to studies done in Ghana [25], Addis Zemen [17] and Wolayitasodo [24]. Such correlation could be because contraceptive usage is strongly not encouraged in Muslim culture, and this in fact avoids unintended pregnancy due to contraceptive failure. Moreover, most Muslim mothers count each child as God’s gift, and these mean that Muslim mothers don’t bother about unintended pregnancy. Furthermore, relative to orthodox Christians, Muslim mothers tended to have higher fertility and greater decision-making power over their wellbeing.

Similar to other studies done in Ethiopia [12, 13, 16, 26], and Kenya [18] the current study at hand revealed that unmarried mothers were at higher odds of unintended pregnancy. The potential reason is that unmarried women may unintentionally participate in sexual activity and this is most likely unwanted if the pregnancy is occurred. Additionally, our culture and community might cause a significant influence on unmarried women not to use contraceptive because of sex is not recommended before the mother is married.

Consistent with other studies conducted in Ethiopia [12, 29] and Nigeria [28] unintended pregnancy is significantly associated with household size, in which mothers from the household size of four and above had higher odds of unintended pregnancy. This might be due to mothers with higher household size might be busy in caring their family and this affects their getting of information and accessing and utilization of maternal health services such as contraceptive methods which in turn end up with unintended pregnancy.

Moreover in this study multiparous and grand multiparous mothers had higher odds of unintended pregnancy and this is congruent with other studies conducted in Ethiopia [14, 16, 17], Ghana [22] and Pakistan [21]. The possible explanation might be these women have enough or adequate number of children with a decreasing intention for the next pregnancy or childbirth.

Furthermore, region was an important community-level variable and mothers from metropolitan and large peripheral regions had higher odds of unintended pregnancy. This regional variation indicated in this study is in line with studies conducted in Ethiopia [29], Kenya [18] and Ghana [22]. This is since pregnancies may be appreciated and accepted in small peripheral regions and mothers in this region did not think about unintended pregnancy. But women in metropolitan and large central regions might be busy because of their intention to advance their economic status and mostly their pregnancies are more likely to be unintended.

This study had strength since it was based on nationally representative data with large sample size. The other strength was we used an appropriate statistical approach (multilevel analysis) to accommodate the hierarchical nature of the data. Moreover, since it is based on the national survey data the study has the potential to give insight for policy-makers and program planners to design appropriate intervention strategies both at national and regional levels. However, this study had limitations in that the EDHS survey is mostly based on respondents’ self-report and might have the possibility of recall bias. Again, this study only shows the associations between unintended pregnancy and some important individual-level and community-level factors that is it did not establish causality.

## Conclusion

In this study the prevalence of unintended pregnancy was high. In a multivariable multilevel analysis, both individual and community level variables were significantly associated with unintended pregnancy in Ethiopia. Of individual-level variables younger age group, being not follower of Muslim religion, unmarried, mothers with higher household size, and multiparous and grand multiparous mothers were at higher odds of unintended pregnancy. Among community-level variables; mothers living in large central regions and metropolitan were at higher odds of unintended pregnancy. Therefore, special attention could be taken for these high-risk groups in terms of increasing accessibility and availability of maternal health services and by doing so this devastating health problem (unintended pregnancy) could be decreased.

## Data Availability

All result-based data are available within the manuscript and anyone can access the data set online from www.measuredhs.com.

## Abbreviations

AOR: Adjusted Odds Ratio
CI: Confidence Interval
DHS: Demographic and Health Survey
EAs: Enumeration Areas
EDHS: Ethiopian Demographic and Health Survey
ICC: Intra Class Correlation
MOR: Median Odd Ratio
PCV: Proportional Change in Variance
PHC: Population and Housing census
